# Student Expenses in Residency Interviewing

**Published:** 2017-08-30

**Authors:** Anne Walling, Kari Nilsen, Paul Callaway, Jill Grothusen, Cole Gillenwater, Samantha King, Gregory Unruh

**Affiliations:** 1 School of Medicine-Wichita, Department of Family and Community Medicine, University of Kansas Medical Center; 2 School of Medicine-Wichita, Office of Graduate Medical Education, University of Kansas Medical Center; 3 School of Medicine-Wichita, Wesley Family Medicine Program, University of Kansas Medical Center; 4 School of Medicine-Kansas City, Office of Graduate Medical Education, University of Kansas Medical Center

**Keywords:** medical students, medical residencies

## Abstract

**Background:**

The student costs of residency interviewing are of increasing concern but limited current information is available. Updated, more detailed information would assist students and residency programs in decisions about residency selection. The study objective was to measure the expenses and time spent in residency interviewing by the 2016 graduating class of the University of Kansas School of Medicine and assess the impact of gender, regional campus location, and primary care application.

**Methods:**

All 195 students who participated in the 2016 National Residency Matching Program (NRMP) received a 33 item questionnaire addressing interviewing activity, expenses incurred, time invested and related factors. Main measures were self-reported estimates of expenses and time spent interviewing. Descriptive analyses were applied to participant characteristics and responses. Multivariate analysis of variance (MANOVA) and chi-square tests compared students by gender, campus (main/regional), and primary care/other specialties. Analyses of variance (ANOVA) on the dependent variables provided follow-up tests on significant MANOVA results.

**Results:**

A total of 163 students (84%) completed the survey. The average student reported 38 (1–124) applications, 16 (1–54) invitations, 11 (1–28) completed interviews, and spent $3,500 ($20–$12,000) and 26 (1–90) days interviewing. No significant differences were found by gender. After MANOVA and ANOVA analyses, non-primary care applicants reported significantly more applications, interviews, and expenditures, but less program financial support. Regional campus students reported significantly fewer invitations, interviews, and days interviewing, but equivalent costs when controlled for primary care application. Cost was a limiting factor in accepting interviews for 63% and time for 53% of study respondents.

**Conclusions:**

Students reported investing significant time and money in interviewing. After controlling for other variables, primary care was associated with significantly lowered expenses. Regional campus location was associated with fewer interviews and less time interviewing. Gender had no significant impact on any aspect studied.

## Introduction

**Figure 1:**
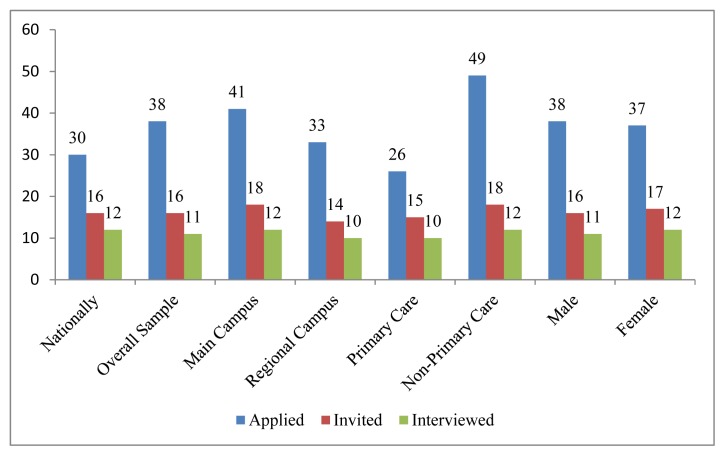
Average number of applications, invitations, and completed interviews by gender, campus type, and primary care application. Note: National data are 2015 NRMP report: study data are 2016.

The National Resident Matching Program (NRMP) increasingly is challenging for participants and disruptive of the senior year of medical education.^1^–^4^ In 2016, 18,187 students of US allopathic medical schools were among 35,476 active applicants for 27,860 Post Graduate Year (PGY) 1 positions.^5^ In 2015, the average successful US allopathic student reported applying to 30 programs, receiving 16 interview invitations, and completing 12 interviews (Figure 1).^6^ The US seniors who did not match submitted an average of 54 applications, received six invitations, and completed six interviews. Individual students submitted an average of 1 – 67 applications depending on specialty.

Interviewing costs for US students have not been reported extensively. Surveys have included graduates of one state,^7^ selected institutions,^2^ a regional campus,^8^ and applicants to specific specialties.^9^–^14^ The two largest studies had response rates of 20% and 47% respectively.^2^,^7^ Low response rates potentially increase the selection bias of surveying specific groups. Total student costs ranged from under $100 to over $20,000 depending on the types of student and scope of costs studied. Several studies have reported lower costs for primary care applicants but other variables influencing cost and time have not been identified.^2^,^8^

This study examined the financial and time costs for residency interviewing of a large class of students and the influence of cost and time in interviewing decisions. The primary objective was to generate information to assist in advising students, inform residency programs, and contribute to curricular planning for the final year. We also were interested in identifying any differences between male and female students’ experiences and any influence of regional campus location. The primary care mission of the regional campuses was expected to lower costs, but this could be countered by the increased distances from major cities and generally higher air fares from regional sites.

## Methods

All fourth-year students of the University of Kansas School of Medicine (KUSM) who participated in NRMP during 2016 were surveyed immediately following announcement of NRMP results. The survey questionnaire was distributed by e-mail weekly for four weeks. Class leaders sent social media reminders two to three times weekly encouraging students to complete the questionnaire. As an incentive, a donation proportional to the response rate was offered to each campus graduation celebration fund.

The 33-item questionnaire was based on a 2015 study conducted on the KUSM Wichita campus,^8^ literature reviews,^1^–^3^,^5^–^12^ and input from faculty, residents, and students. The questionnaire addressed the number, specialty, and location of programs, variables influencing interview choices, cost and time of interviewing, sources of funding of interviews, and any costs covered by programs. The questionnaire included opportunities for narrative comments on specific items and the overall interviewing process. The instrument was pilot-tested by eight students who participated in early match processes. Minor changes were made to four questions to clarify meaning and avoid potential ambiguity.

Descriptive analyses provided details about the students and their survey responses. Chi-square tests were used to determine if there were any statistical differences by specialty choice (primary care versus non-primary care), gender (male and female), as well as campus location (main and regional). This test was chosen because it is used to compare observed frequencies to expected frequencies. *T*-tests were used to compare the average costs of interviewing by specialty choice (primary care versus non-primary care) and campus location (main and regional). This test was selected for these variables because they only have two levels, and the differences between the two levels were of interest. Multivariate analysis of variance (MANOVA) tests were used for simultaneous comparisons between students by gender, campus, and application to primary care (defined as all family medicine, internal medicine, pediatrics, and medicine/pediatrics programs). Analyses of variance (ANOVA) on the dependent variables were conducted as follow-up tests to all significant MANOVA results. Using the Bonferroni Method, each ANOVA was tested at the .025 level. MANOVAs were used because they are able to control for any correlations between dependent variables, while testing for significance between multiple groups.

This study was approved by the University of Kansas Institutional Review Board.

## Results

### Participants

Of 195 eligible students, 163 (84%) completed the questionnaire. The response rates were 78% (94/120) from the Kansas City campus, 91% (61/67) from the Wichita regional campus, and 100% (8/8) from the Salina regional campus. The mean respondent age was 28 (range 24–55) years and 130 (80%) were white (Table 1). Of the 32 non-responders, 26 were male (81%), and 26 were from the main campus (81%).

Seventy-six students (47%) applied to primary care programs. The percentage of primary care applicants was higher for women (52%) than men (42%) but not statistically significant (χ^2^(1, N = 163) = 0.18, *p* = .21). Similarly, the percentage of regional campus students applying to primary care (55%) was not significantly higher than the main campus (43%; χ^2^(1, N = 163) = 0.11, *p* = .15).

### Volume of interviewing

Students applied to an average of 38 programs (range 1–124), received 16 interview invitations (range 1–54), and completed eleven interviews (range 1–28; Figure 1). One hundred and fifty-eight students (98%) interviewed out-of-state, covering 42 states, including Alaska.

A MANOVA to determine the effect of gender, campus (main or regional), and primary care on the five dependent variables related to the volume of interviewing (i.e., the numbers of applications, interview invitations and completions; and cost and time as limiting factors in interview decisions) found no significant difference for gender (Wilks’s Λ = .98, *F*(5,147) = .73, *p* = .60, η^2^ = .02), but significant differences between regional and main campuses (Wilks’s Λ = .89, *F*(5,147) = 3.75, *p* = .003, η^2^ = .11) and between primary care and non-primary care applicants (Wilks’s Λ = .75, *F*(5,147) = 9.8, *p* < .001, η^2^ = .25; Table 2).

The ANOVA by campus type found no significant difference on total number of applications submitted (*F*(1,151) = 2.78, *p* = .09, η^2^ = .02), cost as a limiting factor (*F*(1,151) = .86, *p* = .35, η^2^ = .01), or time as a limiting factor in deciding to interview (*F*(1,151) = 1.9, *p* = .17, η^2^ = .01). Students from the main campus were invited to significantly more interviews (*F*(1,151) = 10.6, *p* = .001, η^2^ = .06), and completed more interviews (*F*(1,151) = 12.9, *p* < .001, η^2^ = .08). The ANOVA found no significant difference for primary care application in cost as a limiting factor (*F*(1,151) = .52, *p* = .47, η^2^ = .003) or time as a limiting factor in deciding to interview (*F*(1,151) = .06, *p* = .80, η^2^ < .001). Non-primary care applicants applied to significantly more programs (*F*(1,151) = 47.7, *p* < .001, η^2^ = .24), and completed more interviews (*F*(1,151) = 9.2, *p* = .003, η^2^ = .06), but were not offered more interviews than primary-care applicants (*F*(1,151) = 3.5, *p* = .06, η^2^ = .02; Table 2).

### Financial costs of interviewing

The average reported cost for interviewing was $3,500 (range $20–$12,000; Tables 3 and 4). On all campuses, applicants to primary care reported an average of about $1,400 less than their classmates applying to other specialties (Table 3). Twenty-two percent of all students spent less than $1,000. However, 35% of primary care applicants reported costs less than $1,000, compared to only 11% of those applying to other specialties (*p* < .001; Table 4).

A MANOVA conducted to determine the effect of gender, campus location, and primary application on the five dependent variables related to financial cost of interviewing (i.e., total estimated expenses, the number of funding sources used, and any contribution from residency programs to travel, lodging, and meal expenses) found no significant difference for gender (Wilks’s Λ = .98, *F*(5,129) = .57, *p* = .72, η^2^ = .02) or campus (Wilks’s Λ = .98, *F*(5,129) = .48, *p* = .82, η^2^ = .02), but a significant difference between primary care and non-primary care application (Wilks’s Λ = .79, *F*(5,129) = 6.83, *p* < .001, η^2^ = .21; Table 2).

An ANOVA on the dependent variables found a significant difference in total estimated expenses between primary care and non-primary care applicants (*F*(1,133) = 11.9, *p* = .001, η^2^ = .08). Students reported using the same funding sources (principally student loans, credit cards, gifts from family members), but non-primary care applicants reported using significantly more funding sources (*F*(1,133) = 3.9, *p* = .05, η^2^ = .03). Students reported a wide range of program financial contributions. Ninety-two percent reported any assistance with payment for meals and 84% reported any contribution to lodging. Only 18% reported any assistance with travel. Primary care applicants were significantly more likely to report any contributions to travel expenses (*F*(1,133) = 4.1, *p* = .05, η^2^ = .03) and lodging (*F*(1,133) = 14.8, *p* < .001, η^2^ = .1), but no significant difference was demonstrated for meals (*F*(1,133) = 1.8, *p* = .16, η^2^ = .01). For each category, the expenses covered by individual programs ranged from zero to all expenses (Table 2).

### Interview time and scheduling

Students reported an average of 26 days spent interviewing (range 1–90). November was the most common month with an average 4.2 interviews per student. Students reported an average 13 days’ notice for interviews (range 1–60 days). All students perceived pressure to respond quickly to interview invitations: 36% responded within 10 minutes and 72% within the hour. Only 5% waited more than 24 hours.

A MANOVA to determine the effect of gender, campus location, and primary care application on five dependent variables related to interview time and scheduling (i.e., days interviewing, number of cancelled and rescheduled interviews, prior notice, and responding to interview invitations within ten minutes) found no significant difference for gender (Wilks’s Λ = .91, *F*(5,105) = 2.03, *p* = .08, η^2^ = .09) or for primary care (Wilks’s Λ = .91, *F*(5,105) = 2.2, *p* = .06, η^2^ = .09). A significant difference was found between regional and main campuses (Wilks’s Λ = .87, *F*(5,105) = 29, *p* = .01, η^2^ = .13; Table 2).

The ANOVA found significantly more days devoted to interviewing for the main campus (*F*(1,109) = 12.7, *p* = .001, η^2^ = .10) and a significantly higher proportion of students responding within ten minutes of interview invitation (*F*(1,109) = 4.63, *p* = .03, η^2^ = .04). No significant campus differences were found for the number of cancelled interviews (*F*(1,109) = 1.09, *p* = .29, η^2^ = .01), rescheduled interviews, (*F*(1,109) = .55, *p* = .46, η^2^ = .01), or length of notice for each interview (*F*(1,109) = 1.14, *p* = .29, η^2^ = .01).

## Discussion

This study provided a detailed picture of the residency interviewing experience of a large class of students at a tri-campus, Midwestern state medical school. The high response rate reflects student leadership and interest in the topic. The key findings are confirmation of the major impact of primary care specialty choice, the modest differences for regional campus students, and the absence of significant gender differences. Average expenses for non-primary care applicants were over 60% higher than those of primary care applicants. When adjusted for specialty choice, regional campus students did not report higher costs despite the distance of regional campuses from major cities. The absence of gender differences may reflect the similarity in specialty choice by KUSM male and female students (e.g., 17% of women and 19% of men applied to surgical specialties).

Similar to other studies^2^,^7^–^14^, our students spent an average $3,500 but the range was $20 to $12,000 and 22% of all students (and 35% of primary care applicants) spent less than $1,000. Students added expenses to existing debt, often using multiple funding sources. The topic of program financial support merits further study as programs compete for the best applicants; for example, nationally in 2016, Internal Medicine program directors reported receiving an average of 2,619 applicants and interviewing 201 applicants.^15^

The time spent interviewing has been reported in one previous study.^11^ This study estimated a median of 20 days for applicants to urology programs in 2006. Time was a limiting factor in interviewing for 58% of students. A few students reported extremely high values, up to 90 days. Students may have under-reported time due to regulations about absences from fourth year courses. Time lost from education is of major concern. In addition to absences, we cannot estimate the negative educational impact of students distracted from their studies by concerns about the match process. Student narrative comments described significant stress over obtaining and completing interviews, especially those involving frequent schedule changes. Students reported that receiving interview invitations in distant cities with only one to two days’ notice was not uncommon, especially late in the interviewing period. Many students experienced constant vigilance and a sense of urgency in decisions about interviewing.

Primary care students received interview invitations from about 60% of applications. Those applying to non-primary care averaged invitations from 37% of applications despite applying to significantly more programs. Both groups of students failed to complete interviews for about one third of invitations. Further research is indicated into the reasons for declining or cancelling interviews, but 63% of our students reported limited interviewing because of financial concerns.

The study has several limitations, including being conducted in a single institution, self-reported costs, possible recall bias, and the definition of primary care that includes students intending to subspecialize. Similarly, grouping surgical specialties masks differences among different specialties. We were unable to include measures of student “competitiveness” in our analysis without compromising anonymity. Application of our findings to other schools must be individualized.

To the extent that our findings are generalizable to other institutions, they provide financial data to the debate over the escalating number of NRMP applications per student. This trend raises costs and strains resources for students and programs despite a sustained match rate for US allopathic graduates of around 94%.^1^,^5^,^16^–^21^ Our report also draws attention to the days lost from education in the fourth year of medical school at a time when national curricular innovations increasingly emphasize the crucial role of the final year in achieving competencies and transitioning to the residency stage of education.

Further studies are indicated in the role of cost and other factors in the failure to complete interviews after invitation, and into the extent and significance of program financial contributions to student interviewing expenses.

## Figures and Tables

**Table 1:** Study participants.

	Respondents (%) (N = 163)	
Sex		
Female	79 (49)	
Male	84 (52)	
Race		
White	130 (80)	
Asian	19 (12)	
Black	6 (4)	
Other or missing	8 (5)	
Campus		
Main	94 (58)	
Regional	69 (42)	
Specialty of Application		
Primary Care	76 (47)	31 (19%) family medicine 28 (17%) internal medicine 13 (8%) pediatrics 4 (2.5%) medicine-pediatrics
Non-Primary Care	87 (53)	35 (22%) surgical specialties 10 (6%) anesthesiology 9 (6%) obstetrics/gynecology 7 (4%) radiology, emergency medicine 6 (4%) psychiatry 3 (2%) dermatology, neurology, pathology 1 (0.6%) preventive medicine, pediatric neurology, Other

**Table 2:** Significant ANOVAs by MANOVA variable.

MANOVA	Significant ANOVA							
	Campus				Primary Care Application			
	Main (N = 94)		Regional (N = 69)		Yes (N = 78)		No (N = 85)	
**Application Activity**								
Applications					26 ± 15.07	n = 76	48.8 ± 23.83	n = 83
Interview offers	18.35 ± 9.8	n = 93	13.62 ± 8.36	n = 66				
Completed interviews	12.17 ± 4.59	n = 93	9.58 ± 4.0	n = 66	9.89 ± 4.06	n = 76	12.19 ± 4.68	n = 83
**Financial Costs ($)**								
Total spent interviewing					2825 ± 2422	n = 69	4254.51 ± 2446.59	n = 72
Number of funding sources					1.6 ± .66	n = 69	1.9 ± .72	n = 72
Any travel paid					1.26 ± .44	n = 69	1.1 ± .31	n = 72
Any lodging paid					1.95 ± .21	n = 69	1.7 ± .46	n = 72
**Interview Time and Scheduling**								
Days interviewing	28.17 ± 16.23	n = 69	19.21 ± 8.25	n = 48				
Interview offer response <10 mins	1.6 ± 0.49	n = 69	1.81 ± 0.39	n = 48				

**Table 3:** Estimated average total interviewing expenses ($) by campus and primary care application.

	Main Campus	Regional Campuses	All Students	*p* value
**Estimated average costs (range)**	3,652 (20 – 11,000)	3,342 (75 – 12,000)	3,516 (20 – 12,000)	0.461
**Primary care applicants (range)**	2,827 (20 – 7,000)	2,701 (75 – 12,000)	2,765 (20 – 12,000)	0.822
**Non-primary care applicants (range)**	4,305 (100 – 11,000)	4,087 (300 – 10,000)	4,219 (100 – 11,000)	0.713

**Table 4:** Estimated total interviewing expenses.

Amount Spent	Primary Care	Non-Primary Care	*p* value
			
$≤1,000	26 (35%)	9 (11%)	< .001
$1,001 – 5,000	39 (53%)	51 (65%)	< .001
$5,001 – 10,000	7 (10%)	18 (23%)	< .001
>$10,000	2 (3%)	1 (1%)	0.06
